# Supplementary material for: Weight gain after renal transplant: Incidence, risk factors, and outcomes
Source: PLoS One. 2022 Jun 2;17(6):e0268044. doi: 10.1371/journal.pone.0268044 (PMC9162351; doi:10.1371/journal.pone.0268044)
Supplement: Attachment — Submitted filename: Response to Reviewers..docx [file pone.0268044.s001.docx]

Response to Reviewers

1. *It’s unclear to me that weight gain was observed in 235 patients and weight decreased in 57 patients but Table 1 shows gained weight (n=161) and stable weight (n=134).*

- Agree, typing errors.

- Corrected in manuscript.

2. *How did the authors choose a BMI >2 as a cutoff? Do the authors have literature support this cutoff?*

- Definitions of clinically significant weight change are not consistent in the literature. Some studies used weight changes more than 5% or 7 % but we decide to choose more conservative measure of a BMI >= 2 kg/m^2^ gain.
- Verma S, Liew A, Subramaniam M, Poon LY. Effect of treatment on weight gain and metabolic abnormalities in patients with first-episode psychosis. *Aust N Z J Psychia.* 2009;43(9):812–7.
- Knowler WC, Barrett-Connor E, Fowler SE, et al. Reduction in the incidence of type 2 diabetes with lifestyle intervention or metformin. *N Engl J Med.* 2002;346(6):393–403.

3. *Table 1 should have legend explaining the test comparing the two groups (i.e. Fisher’s exact test and/or unpaired t-test). Also Table 2 and Table 3.*

**Table 1.** Baseline characteristics

| Characteristic  N | Total  295 | Gained Weight  161 | Stable Weight  134 | P value |
| --- | --- | --- | --- | --- |
| Age ^a^ | 45.3±15.1 | 45.0±14.6 | 45.7±15.7 | 0.751 |
| Gender ^b^ |  |  |  |  |
| Female | 119 | 71 | 48 | 0.155 |
|  | 40.30% | 44.10% | 35.80% |  |
| Male | 176 | 90 | 86 |  |
|  | 59.70% | 55.90% | 64.20% |  |
|  |  |  |  |  |
| Non-Smoker ^b^ | 258 | 139 | 119 | 0.262 |
|  | 87.50% | 86.30% | 88.80% |  |
| Ex-smoker | 13 | 10 | 3 |  |
|  | 4.40% | 6.20% | 2.20% |  |
| Smoker | 24 | 12 | 12 |  |
|  | 8.10% | 7.50% | 9.00% |  |
| DM ^b^ | 77 | 44 | 33 | 0.689 |
|  | 26.30% | 27.50% | 24.80% |  |
| HTN ^b^ | 234 | 129 | 105 | 0.885 |
|  | 79.60% | 80.10% | 78.90% |  |
| CAD ^b^ | 39 | 19 | 20 | 0.490 |
|  | 13.40% | 11.90% | 15.20% |  |
| Bariatric ^b^ | 12 | 8 | 4 | 0.557 |
|  | 4.10% | 5.00% | 3.00% |  |
| Duration of dialysis ^a^ | 2.3±2.4 | 2.0±2.2 | 2.6±2.6 | 0.032 |
| ECOG ^b^ |  |  |  |  |
| 0 | 251 | 143 | 108 | 0.083 |
|  | 86.30% | 89.90% | 81.80% |  |
| 1 | 32 | 14 | 18 |  |
|  | 11.00% | 8.80% | 13.60% |  |
| 2 | 8 | 2 | 6 |  |
|  | 2.70% | 1.30% | 4.50% |  |
| Donor ^b^ |  |  |  |  |
| Living | 227 | 131 | 96 | 0.053 |
|  | 76.90% | 81.40% | 71.60% |  |
| Deceased | 68 | 30 | 38 |  |
|  | 23.10% | 18.60% | 28.40% |  |
| Induction ^b^ |  |  |  |  |
| ATG | 185 | 98 | 87 | 0.546 |
|  | 62.70% | 60.90% | 64.90% |  |
| Basiliximab | 110 | 63 | 47 |  |
|  | 37.30% | 39.10% | 35.10% |  |

^a^ The P value was calculated by the unpaired t-test.

^b^ The P value was calculated by the Fisher's exact test.

**Table 2.** Metabolic & laboratory variables:

| Characteristic | Total | Gained Weight | Stable Weight | P value |
| --- | --- | --- | --- | --- |
| BMI ^a^ |  |  |  |  |
| Pre | 26.0±5.2 | 25.5±5.1 | 26.7±5.2 | 0.051 |
| Post 1 month | 26.0±5.2 | 26.0±5.3 | 26.0±5.2 | 0.989 |
| Post 6 months | 27.5±5.3 | 28.4±5.2 | 26.3±5.1 | 0.001 |
| Post 12 months | 28.6±5.6 | 30.7±5.5 | 26.7±5.2 | <0.001 |
| Creatinine umol/L ^a^ |  |  |  |  |
| 1 week | 111.5±65.1 | 105.3±55.3 | 118.8±74.7 | 0.077 |
| 1 month | 102.3±36.8 | 98.0±34.2 | 107.3±39.2 | 0.031 |
| 6 months | 97.9±33.1 | 92.8±29.0 | 103.9±36.6 | 0.004 |
| 12 months | 93.5±29.7 | 90.7±26.5 | 96.9±33.0 | 0.073 |
| eGFR ml/min ^a^ |  |  |  |  |
| 1 week | 73.6±27.9 | 76.1±28.6 | 70.6±26.9 | 0.089 |
| 1 months | 73.9±22.5 | 76.5±22.8 | 70.8±21.73 | 0.031 |
| 6 months | 75.8±21.1 | 78.4±20.7 | 72.7±21.2 | 0.020 |
| 12 months | 79.5±20.6 | 80.4±20.2 | 78.4±21.1 | 0.402 |
| Proteinuria ^b^ |  |  |  |  |
| 1 month | 88 | 46 | 42 | 0.612 |
|  | 29.80% | 28.60% | 31.30% |  |
| 6 months | 80 | 36 | 44 | 0.049 |
|  | 27.10% | 22.40% | 32.80% |  |
| 12 months | 72 | 37 | 35 | 0.587 |
|  | 24.40% | 23.00% | 26.10% |  |
| HbA1c ^b^ |  |  |  |  |
| <5.7% | 189 | 100 | 89 | 1 |
|  | 93.60% | 93.50% | 93.70% |  |
| 5.7%-6.9% | 32 | 17 | 15 | 1 |
|  | 71.10% | 70.80% | 71.40% |  |
| >6.9% | 59 | 35 | 24 | 0.763 |
|  | 81.90% | 83.30% | 80.00% |  |
| HbA1c (%) ^a^ |  |  |  |  |
| 3 months | 6.4±1.6 | 6.4±1.6 | 6.4±1.6 | 0.909 |
| 6 months | 6.7±1.7 | 6.6±1.6 | 6.7±1.8 | 0.656 |
| 12 months | 6.8±1.8 | 6.8±1.8 | 6.8±1.7 | 0.825 |
| FBS (mg/dl) ^b^ |  |  |  |  |
| <5.5 | 165 | 85 | 80 | 0.091 |
|  | 92.20% | 88.50% | 96.40% |  |
| 5.5-7 | 48 | 25 | 23 | 0.123 |
|  | 77.40% | 69.40% | 88.50% |  |
| >7 | 67 | 39 | 28 | 0.228 |
|  | 82.70% | 78.00% | 90.30% |  |
| FBS (mg/dl) ^a^ |  |  |  |  |
| 1 month | 7.0±3.6 | 7.3±4.1 | 6.7±2.8 | 0.188 |
| 6 months | 7.0±3.4 | 7.1±3.4 | 6.9±3.4 | 0.498 |
| 12 months | 6.8±3.3 | 7.0±3.6 | 6.6±2.9 | 0.369 |
| LDL (mg/dl) ^a^ |  |  |  |  |
| Pre | 2.6±0.8 | 2.6±0.8 | 2.5±0.8 | 0.294 |
| 3 months | 2.5±0.9 | 2.5±0.8 | 2.6±1.0 | 0.602 |
| 12 months | 2.5±0.9 | 2.5±0.7 | 2.5±1.0 | 0.869 |

^a^ The P value was calculated by the unpaired t-test.

^b^ The P value was calculated by the Fisher's exact test.

**Table 3.** Complications:

| Characteristic | Total | Gained Weight | Stable Weight | P value |
| --- | --- | --- | --- | --- |
| Rejection ^a^ | 29 | 18 | 11 | 0.437 |
|  | 9.80% | 11.20% | 8.20% |  |
| DGF ^a^ | 18 | 9 | 9 | 0.808 |
|  | 6.10% | 5.60% | 6.80% |  |
| Wound infection ^a^ | 6 | 4 | 2 | 0.693 |
|  | 2.00% | 2.50% | 1.50% |  |
| Urine leak ^a^ | 3 | 2 | 1 | 1 |
|  | 1.00% | 1.20% | 0.80% |  |
| UTI ^a^ | 79 | 41 | 38 | 0.599 |
|  | 26.80% | 25.50% | 28.40% |  |
| NODAT ^a^ | 14 | 7 | 7 | 0.787 |
|  | 4.80% | 4.30% | 5.30% |  |
| CMV ^a^ | 158 | 75 | 83 | 0.007 |
|  | 53.70% | 46.60% | 62.40% |  |
| BK ^a^ | 52 | 29 | 23 | 0.880 |
|  | 17.70% | 18.00% | 17.30% |  |
| Arrhythmia ^a^ | 7 | 2 | 5 | 0.251 |
|  | 2.40% | 1.20% | 3.70% |  |
| ACS ^a^ | 3 | 0 | 3 | 0.093 |
|  | 1.00% | 0.00% | 2.20% |  |
| Mortality ^a^ | 14 | 6 | 8 | 0.418 |
|  | 4.70% | 3.70% | 6.00% |  |
| Started insulin ^a^ | 8 | 5 | 3 | 0.732 |
|  | 2.70% | 3.10% | 2.20% |  |

^a^ The P value was calculated by Fisher's exact test.

4. *What were the maintenance immunosuppression in each group? This data should be included. Was the weight gain driven by prednisone? Are differences in other regimens?*

- All patients had the *same* tacrolimus-based maintenance immunosuppression regimen which include Prednisolone, Tacrolimus and mycophenolate.
- *Clarified in revised manuscript.*

5. *Is the repeated measured analysis with contrasts? Can you please explain more detail in the methods how this was done since you are comparing groups at different time points?*

- We applied general linear model repeated measures ANOVA with three time points, baseline, 6 months, and 12 months to determine the intergroup differences (between-group effect for those who gained weight those who did not). Nevertheless, all within-group, between-group, and their interaction were not significant. In addition, we have compared the two groups in each time point separately in Table 2. That is why we did not conduct post-hoc contrasts.

6. *How did you define proteinuria? Did you quantify the proteinuria with a Urine P/C ratio?*

- Proteinuria measured by using spot urine albumin creatinine ratio (ACR). Proteinuria is urine ACR > 3 mg/mmol.
- Definitions added to revised Manuscript.

7. *More detail is needed in Table 3: how did you define rejection, DGF, CMV, and BK? How long was the follow up for patients, was this all at 1 year?*

- Definitions added to revised Manuscript.

8. *In the multivariable logistic regression, how did you choose the variables that went into the multivariable analysis? Did you do a univariate analysis and those significant went into the multivariable analysis? How did you come to choose age > 40 rather than continuous variable?*

- We think that categorizing the age variable would make it more representable and easier to comprehend. The cutoff point of 40 years was selected arbitrarily after the trial of several other numbers (30, 45, 50, and 60).
- Clinically important variables were used to build multivariate logistic regression model. The final model was selected after conducting backward stepwise selection method to choose the best fit model.

9. *The authors note mortalities? Did this occur in the first year? If so, how was the weight account for if the patient died before 1 year?*

- Yes, 1^st^ year mortality, we used last available weight.

10. Figure 1 and Figure 2 need legends and should be separated.

- OK, done

11. *Abbreviations and acronyms such as CMV, BK, DM, FBS .*

*In Table 1, please report units of measurement for “Duration of dialysis” (e.g. months or years)*

*In Figures 1 and 2, please report units of measurement for blood pressure. In Figure 2, please label the two groups appropriately by adding “BMI” to “<2” and “>=2”.*

*In Table 2, please clarify criteria for “proteinuria”.*

- All corrected in revised manuscript.

12. *In Table 2, “FBS” is reported as mg/dL, however the values provided appear to be consistent with mmol/L.*

- corrected in revised manuscript

13. *In Table 3, please define “BK”. For instance, is this referring to BKV nephropathy, BKV viremia or BKV viruria?*

- BK Viremia, corrected in revised manuscript.

14. *The authors report CMV infection was significantly less common in the group that gained more weight, and they could not find an explanation for this finding. However, they do not discuss any further analysis that may have been done. It may be useful to explore variables such as CMV serostatus of the donor/recipient pair, CMV prophylaxis regimen, and donor type (there was a trend toward more deceased donors in the "stable weight" group)*

- in our cohort, all our recipients are seropositive for CMV IgG and they maintained on Valganciclovir 450 mg daily for 3 months’ post-transplant as a standard protocol in our center.

15. *what does "bariatric" refer to?*

- Gastric sleeve.

16. *What is the unit used to express duration of dialysis?*

- Years.

17. *What does DM, HTN and CAD stand for?*

Diabetes Mellitus, Hypertension, Coronary artery disease.

Clarified in revised manuscript

18. *Similarly, table 2 also needed to be revised to correct for incomplete descriptions and units.*

*What was the definition of CMV infection used for the manuscript?*

- CMV Viremia which mean positive CMV PCR. CMV PCR done in 1^st^ year as a survilance on monthly bases for 1^st^ 6 months then at 9 & 12 months.
- All corrected in revised manuscript

Thank you .
