# Supplementary material for: Weight gain after renal transplant: Incidence, risk factors, and outcomes
Source: PLoS One. 2022 Jun 2;17(6):e0268044. doi: 10.1371/journal.pone.0268044 (PMC9162351; doi:10.1371/journal.pone.0268044)
Supplement: Attachment — Submitted filename: Responses to reviewers.docx [file pone.0268044.s002.docx]

Responses to reviewers:

- Reference list has been review again.

1) Also please put the details in the methods/results on what was done specifically for the anova testing, as described in the response letter.

Done .

2) Also please put the details in the methods/results on what was done specifically for the multivariable logistic regression, as described in the response letter.

Done

3) The manuscript needs thorough editing for fluency and grammar as also indicated by Reviewer 1.

Done

4) Manuscript has been reviewed and edited again for typing and grammatical errors.

Thank you
